# Reliability and validity of the Swedish indicator ‘Drugs that should be avoided in older people’—an appraisal of a set of potentially inappropriate medications

**DOI:** 10.1007/s00228-024-03700-x

**Published:** 2024-05-14

**Authors:** Naldy Parodi López, Staffan A. Svensson, Johan Lönnbro, Mikael Hoffmann, Susanna M. Wallerstedt

**Affiliations:** 1 Department of Pharmacology, Sahlgrenska Academy, University of Gothenburg, Gothenburg, Sweden; 2 Department of Clinical Pharmacology, Sahlgrenska University Hospital, Gothenburg, Sweden; 3 Nötkärnan Bergsjön Health Centre, Gothenburg, Sweden; 4 Department of Internal Medicine, Sahlgrenska University Hospital, Gothenburg, Sweden; 5 NEPI Foundation – Swedish Network for Pharmacoepidemiology, Linköping University, Linköping, Sweden; 6 HTA-Centrum, Sahlgrenska University Hospital, Gothenburg, Sweden

**Keywords:** Aged, Older people, Inappropriate prescribing, Potentially inappropriate medications, Swedish indicator

## Abstract

**Purpose:**

To analyse the reliability and validity of the Swedish indicator ‘Drugs that should be avoided in older people’.

**Methods:**

From a previous study that included consecutive primary care patients ≥ 65 years of age, all patients ≥ 75 years of age were analysed. Two physicians independently screened their medication lists and medical records, applying the Swedish indicator which includes potentially inappropriate medications (PIMs): long-acting benzodiazepines, drugs with anticholinergic action, tramadol, propiomazine, codeine, and glibenclamide. The clinical relevance of identified PIMs was independently assessed. Thereafter, the physicians determined in consensus whether some medical action related to the drug treatment was medically justified and prioritised before the next regular visit. If so, the drug treatment was considered inadequate, and if not, adequate.

**Results:**

A total of 1,146 drugs were assessed in 149 patients (75‒99 years, 62% female, 0‒20 drugs per patient). In 29 (19%) patients, at least one physician identified ≥ 1 PIM according to the indicator at issue; 24 (16%) patients were concordantly identified with ≥ 1 such PIM (kappa: 0.89). Of 26 PIMs concordantly identified, the physicians concordantly assessed four as clinically relevant and 12 as not clinically relevant (kappa: 0.17). After the consensus discussion, six (4%) patients had ≥ 1 PIM according to the studied indicator that merited action. Using the area under the receiver operating characteristic (ROC) curve, the indicator did not outperform chance in identifying inadequate drug treatment: 0.56 (95% confidence interval: 0.46 to 0.66).

**Conclusion:**

The Swedish indicator has strong reliability regarding PIM detection but does not validly reflect the adequacy of drug treatment.

**Supplementary Information:**

The online version contains supplementary material available at 10.1007/s00228-024-03700-x.

## Introduction

Over the last decades, drug treatment in older people has received much attention, both in research and in the monitoring of health care quality. Drug treatment challenges in this age group include the presence of multiple diseases that require many medications and pharmacokinetic changes such as gradually declining kidney function and increased sensitivity to medications due to impaired physiologic compensatory mechanisms. In Sweden, more than 2 million people ≥ 65 years of age, 95% of all inhabitants in this age group, filled at least one drug prescription in 2023 [1].

Suboptimal drug treatment in older people is often described in terms of potentially inappropriate medications (PIMs), including, for instance, long-acting benzodiazepines or first-generation antihistamines. Several criteria sets to identify and analyse suboptimal drug treatment have been developed [2]; some are explicit, and others are implicit. Explicit criteria are usually described as drug-specific or disease-specific, while implicit criteria rely on clinical judgement [3]. An early example of an explicit criteria set is the Beers criteria from the United States, introduced in the 1990s [4] and repeatedly updated by the American Geriatrics Society, most recently in 2023 [5]. In Europe, some recognised criteria sets are the EU(7)-PIM list published in 2015 [6] and the Screening Tool of Older Persons’ Prescriptions (STOPP)/Screening Tool to Alert to Right Treatment (START), last updated in 2023, where STOPP describes PIMs and START potential prescribing omissions (PPOs) [7]. In 2004, the Swedish National Board of Health and Welfare introduced a criteria set, described as indicators for the quality of drug therapy in the elderly [8], with the latest update in 2017 [9]. Typically, the choice of PIMs and diagnosis/conditions to include in a criteria set to represent suboptimal drug treatment in older people relies on expert panel opinions [10].

The Swedish criteria set (see supplement) is applicable to people aged 75 years or older. It includes drug-specific and diagnosis-specific criteria. The former are distributed within nine subsets, of which ‘Drugs that should be avoided in older people unless specific reasons exist’ (hereafter called ‘Drugs that should be avoided in older people’) is the focus of this study. This criterion includes the following drugs and drug classes: long-acting benzodiazepines, drugs with anticholinergic action, tramadol, propiomazine, codeine, and glibenclamide [9]. Since 2011, the studied criterion has been used in Sweden as an indicator to monitor health care quality at national and regional levels, labelled as ‘inappropriate drugs’ [11]. Although also used in research [12–15], reliability and validity of this specific indicator have not been addressed in the international literature. In this study, we thus aimed to analyse these aspects.

## Methods

This study was based on data collected in two previous studies that included 302 consecutive patients, aged 65 years or older, who had a scheduled medical visit in either of two primary care centres in Sweden over a 3-week period in 2017 and whose medication has been thoroughly assessed, for instance, by the application of several criteria sets [16, 17]. In the current study, we focused on a subset of the Swedish criteria set, i.e. ‘Drugs that should be avoided in older people’ [9], and the subgroup of patients for whom these are primarily intended, i.e. those who are ≥ 75 years of age.

**Fig. 1:**
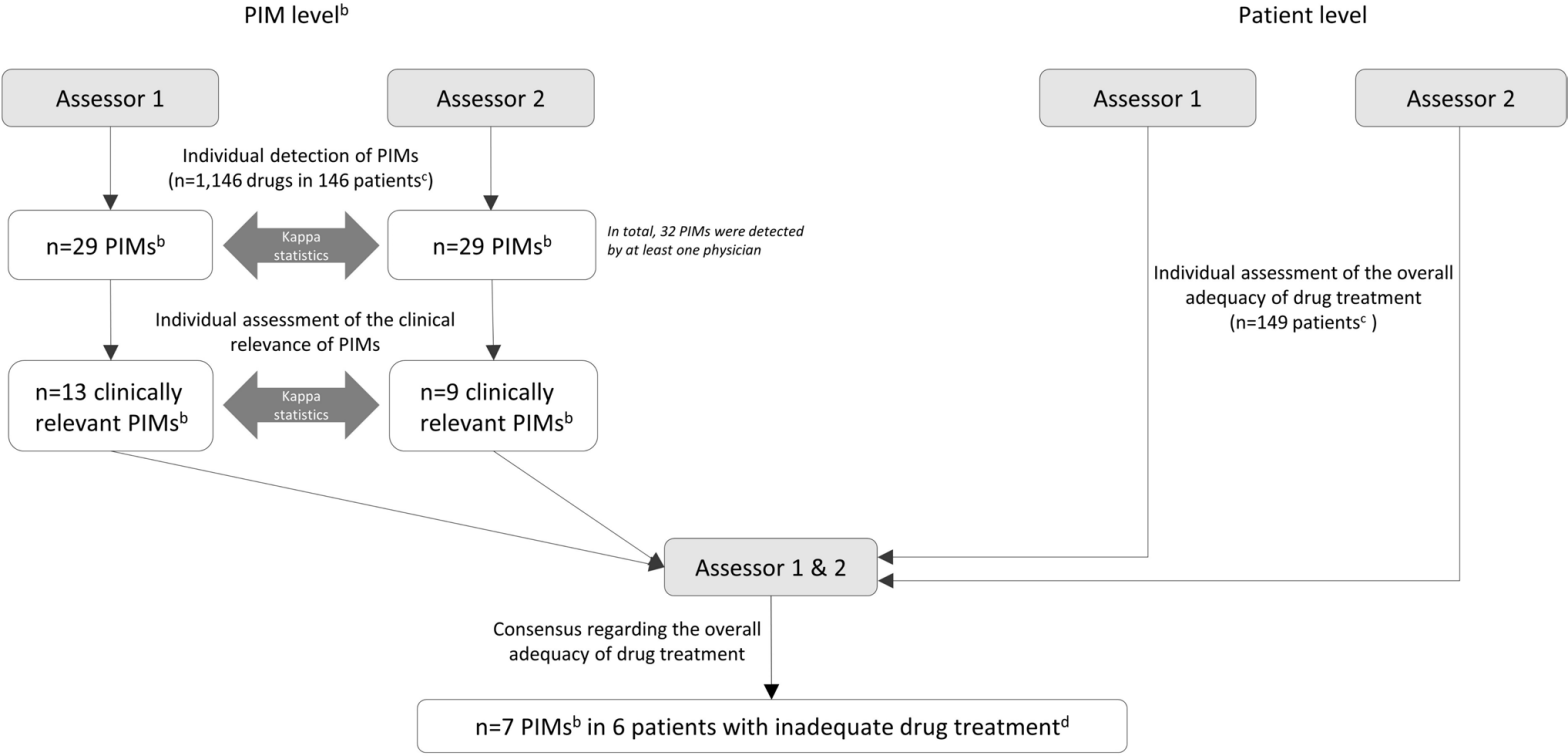
Overview of the assessments performed in the present study^a^, as well as the results. ^a^Detection and clinical relevance assessment of potentially inappropriate medications (PIMs), as well as of potential prescribing omissions (PPOs), was performed systematically in the original studies [16, 17] using three screening tools: STOPP/START [18], EU-7(PIM) list [6], and all PIMs/PPOs included in the Swedish set [9]. ^b^PIMs reported here are restricted to those in the Swedish indicator ‘Drugs that should be avoided in older people’ and among patients aged ≥ 75 years (see Table 5). ^c^There were 149 patients aged ≥ 75 years, of which 146 had drug treatment. ^d^Inadequate drug treatment was defined as follows: one or more actions related to the drug treatment would have been medically justified and prioritised before the next regular visit but were not carried out. The total number of patients with inadequate drug treatment (i.e. not only due to Swedish indicator) was *n* = 48

The process of the assessments performed in the underlying studies is described in Fig. 1. Two specialist physicians (N.P.L., general practitioner; S.A.S., general practitioner/clinical pharmacologist) compiled the patients’ medication based on medical records up to 2½ years before the current medical visit. Based on the medication list and the medical records, they then independently identified PIMs/PPOs according to the STOPP/START criteria version 2 [18], the EU(7)-PIM list [6], and the Swedish criteria set [9] and subsequently assessed their clinical relevance, in one of the following categories: (i) clinically relevant; (ii) of uncertain clinical relevance, but with one or more related medical actions suggested; (iii) not clinically relevant; or (iv) of uncertain clinical relevance, with no related medical action suggested. The two former categories were collapsed into the category ‘clinically relevant’, and the two latter into the category ‘not clinically relevant’. Second, they independently, and then in consensus, categorised the patient’s overall drug treatment at the visit as *adequate* or *inadequate*. Adequate drug treatment reflected that no additional action related to the drug treatment would have been medically justified and prioritised before the next routine visit, such as the regular annual check-up for patients with chronic diseases. Inadequate drug treatment, on the other hand, reflected that one or more actions related to the drug treatment would have been medically justified and prioritised but had not been carried out. Called-for actions could include, for example, retrieving more information about the patient, withdrawing a drug, or ordering a laboratory test.

In the present study, focusing on the subgroup of patients who were ≥ 75 years old and ‘Drugs that should be avoided in older people’, we identified patients treated with medications included in the studied indicator. We recorded medical actions for these PIMs identified by any of the assessors. In cases where the overall drug treatment in consensus was categorised as inadequate, we also recorded whether the related action judged to be medically justified and prioritised concerned a drug included in the abovementioned indicator. When no action was deemed medically justified or prioritised, the underlying reason(s) were noted.

### Statistical analysis

Descriptive analyses were performed using SPSS Statistics for Windows, version 27.0 (IBM Corp., Armonk, NY, USA). To assess the reliability of the studied indicator, inter‐rater agreement at the patient level, for the independent identification of ≥ 1 PIMs in the indicator ‘Drugs that should be avoided in older people’, was estimated using kappa statistics [19]. Based on concordantly identified PIMs, we then calculated the kappa value regarding the physicians’ assessment of their clinical relevance. The inter-rater agreement was interpreted according to the kappa value as follows: none (< 0.20), minimal (0.21–0.39), weak (0.40–0.59), moderate (0.60–0.79), strong (0.80–0.89), and almost perfect (> 0.90) [19]. To evaluate the ability of the studied indicator to classify drug treatment in older people as either adequate or inadequate, we used the area under the receiver operating characteristic (ROC) curve, reflecting the diagnostic capability of a test against a reference standard [20]. The reference standard was the consensus decision by the two specialist physicians, i.e. whether the drug treatment was adequate or inadequate. Finally, we calculated sensitivity and specificity, as well as positive and negative predictive values at the optimal cut-off point, defined as the highest sum of sensitivity and specificity [21]. For comparison, we estimated the area under the ROC curve and the other test accuracy measures based solely on the number of drugs in the medication list.

## Results

In all, 149 patients were included in the study (Table 1). A total of 1,146 drugs were included in the medication lists of 146 patients; three (2%) patients had no prescribed drug treatment.

**Table 1:** Patient characteristics (*n* = 149)

Age, median years (range)		82 (75–99)
Female sex, *n* (%)		93 (62)
Multi-dose drug dispensing, *n* (%)		25 (17)
Nursing home resident, *n* (%)		26 (17)
Substances appearing in ≥ 20% of the medication lists, *n* (%)	Paracetamol	82 (55)
	Furosemide	43 (29)
	Cyanocobalamin	37 (25)
	Acetylsalicylic acid	35 (23)
	Omeprazole	33 (22)
	Simvastatin	33 (22)
	Metoprolol	32 (21)
	Felodipine	32 (21)
	Atorvastatin	30 (20)

In 29 (19%) patients, at least one of the physicians identified ≥ 1 PIM according to the indicator ‘Drugs that should be avoided in older people’ (kappa: 0.89; Fig. 1). Of these patients, 24 (16% of all) were concordantly identified as having ≥ 1 such PIM.

A total of 32 PIMs according to the studied indicator were identified by at least one physician (Table 2), 18 (56%) of which were prescribed on an as-needed basis. Of these 32 PIMs, 26 were concordantly identified by both physicians. Of these 26, in turn, the physicians concordantly assessed four as clinically relevant and 12 as not clinically relevant; the remaining 10 were discordantly assessed regarding the clinical relevance (kappa: 0.17; Fig. 1). The six PIMs that were identified by one physician only concerned drugs that (i) were prescribed as needed (clemastine, *n* = 1; glycopyrronium, *n* = 1; meclozine, *n* = 1; tramadol, *n* = 1); (ii) had an unclear withdrawal decision at the end of the index visit (tramadol, *n* = 1); or (iii) were identified by both physicians as part of another indicator (codeine, *n* = 1).

**Table 2:** Number of PIMs according to the Swedish indicator ‘Drugs that should be avoided in older people unless specific reasons exist’ in 149 patients ≥ 75 years of age with a total of 1,146 drugs in their medication lists after a medical visit to either of two primary care centres. Two specialist physicians’ identification of such PIMs are presented in A, and their assessments regarding the clinical relevance^a^ of concordantly identified PIMs in B

**A**			
	**Assessor A**	**Assessor B**	**Concordant identification**^**b**^
***Total***	***29***	***29***	***26/32***
Long-acting benzodiazepines	7	7	7/7
Drugs with anticholinergic action	12	13	11/14
Tramadol	1	1	0/2
Propiomazine	4	4	4/4
Codeine	4	3	3/4
Glibenclamide	1	1	1/1

**B**			
	**Assessor A**	**Assessor B**	**Concordantly assessed as clinically relevant**^**c**^
***Total***	***11***	***7***	***4/26***
Long-acting benzodiazepines	4	1	1/7
Drugs with anticholinergic action	1	3	0/11
Tramadol	NA	NA	NA
Propiomazine	4	3	3/4
Codeine	1	0	0/3
Glibenclamide	1	0	0/1

*NA* not applicable, *PIM* potentially inappropriate medication

^a^A PIM was categorised as clinically relevant if assessed either as ‘clinically relevant’ or ‘of uncertain clinical relevance, but with one or more related medical actions suggested’

^b^Number of concordantly identified PIMs is presented as numerator, and all PIMs identified by at least one physician as denominator

^c^Number of concordantly identified PIMs that were concordantly assessed as clinically relevant is presented as numerator and all concordantly identified PIMs as denominator

In the consensus discussions, seven of the PIMs according to the studied indicator were considered medically justified and prioritised to act upon before the next regular physician visit: four PIMs were deemed suitable for discontinuation at the physician visit (hydroxyzine, *n* = 2; amitriptyline, *n* = 1; propiomazine, *n* = 1), and three were deemed suitable to withdraw or switch before the next routine visit (nitrazepam, clemastine, codeine/paracetamol, all *n* = 1) (Table 3). The remaining 25 PIMs did not merit action before the next routine visit based on the specific patient’s situation (amitriptyline, *n* = 3; diazepam, *n* = 3; tolterodine, *n* = 3; propiomazine, *n* = 3; codeine/paracetamol, *n* = 3; meclozine, *n* = 2; flunitrazepam, *n* = 2; tramadol, *n* = 2; glycopyrronium, glibenclamide, clomipramine, nitrazepam, all *n* = 1).

**Table 3:** PIMs according to the Swedish indicator ‘Drugs that should be avoided in older people unless specific reasons exist’, identified in 149 patients ≥ 75 years of age after a medical visit to either of two primary care centres, and related actions that would have been medically justified and prioritised but were not acted upon, according to an overall drug treatment assessment performed in retrospect by two physicians in consensus

		***n*** **(%)**^**a**^	**Medically justified and prioritised to act upon during the current visit or before the next routine visit**	
			**Yes, action**	**No action, or action during the next routine visit, reason**
**Long-acting benzodiazepines, total**		**7 (5)**	***n* = 1**	***n* = 6**
	Diazepam	3 (2)	-	No action: rescue treatment in case of seizures in patients with epilepsy (*n* = 2) Action at the next routine visit: switch may be considered, however: low dose, infrequently used, and without reported side effects (*n* = 1)
	Flunitrazepam^b^	2 (1)	-	No action: low dose without reported side effects (*n* = 1) Action during the next routine visit: switch to zopiclone, or an SSRI, may be considered (*n* = 1)
	Nitrazepam^b^	2 (1)	Withdrawal (*n* = 1)	Action at the next routine visit: Switch to zopiclone may be considered (*n* = 1)
**Drugs with anticholinergic action, total**		**14 (9)**	***n* = 4**	***n* = 10**
	Amitriptyline	4 (3)	Withdrawal (*n* = 1)	No action: prescribed for neuropathic pain (*n* = 3)
	Tolterodine	3 (2)	-	No action: attempts to withdraw have been made but the symptoms recurred, prompting restart (*n* = 1); no side effects (*n* = 1) Action at the next routine visit: check indication (*n* = 1)
	Hydroxyzine	2 (1)	Withdrawal (*n* = 2)	-
	Meclozine	2 (1)	-	No action: prescribed by a neurologist against pramipexole’s side effects (*n* = 1); low doses, infrequent use (*n* = 1)
	Glycopyrronium	1 (0.7)	-	No action: palliative patient (*n* = 1)
	Clemastine	1 (0.7)	Withdrawal (*n* = 1)	-
	Clomipramine	1 (0.7)	-	No action: indication: cataplexy, follow-up by neurologist (*n* = 1)
**Tramadol**		**2 (1)**	-	Action at the next routine visit: withdrawal may be considered, however, used as needed (*n* = 2)
**Propiomazine**		**4 (3)**	Withdrawal or switch to zopiclone (*n* = 1)	Action at the next routine visit: withdrawal because of unclear indication, however, low dose (*n* = 1); withdrawal/switch, however, low dose (*n* = 1); withdrawal, however, infrequent use (*n* = 1)
**Codeine**^**c**^		**4 (3)**	Withdrawal (*n* = 1)	No action: low dose, used as needed, and have tried other drugs without effect (*n* = 1); infrequent use, adequate follow-up (*n* = 1) Action at the next routine visit: check indication, however, no overconsumption (*n* = 1)
**Glibenclamide**^**b**^		**1 (0.7)**	-	Action at the next routine visit: withdrawal/switch may be considered, however, no hypoglycaemia and on insulin (*n* = 1)

*PIM* potentially inappropriate medication, *SSRI* selective serotonin reuptake inhibitors

^a^A total of 29 patients were treated with 32 PIMs. No patient was treated with more than one drug within same drug class

^b^Withdrawn from the Swedish market after data collection

^c^Fixed combination with paracetamol

**Fig. 2:**
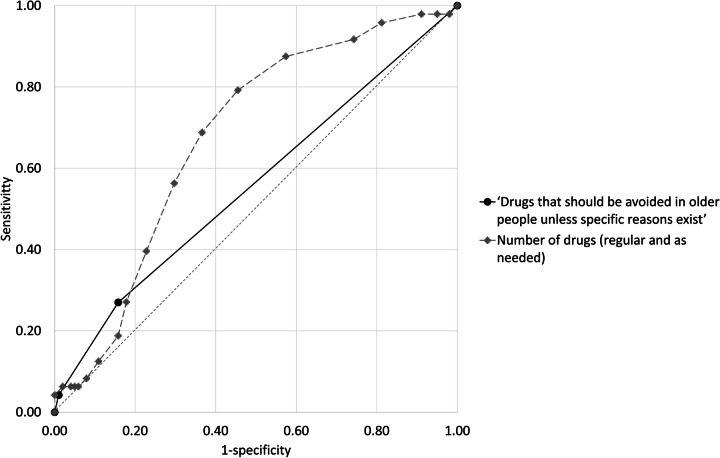
Receiver operating characteristic (ROC) curves for the Swedish indicator ‘Drugs that should be avoided in older people unless specific reasons exist’ to identify the overall drug treatment as adequate or inadequate.^a^ For comparison, a ROC curve based on the number of drugs in the medication list (regular and as needed) is presented. The random classifier is denoted with a dashed line in grey. ^a^Reference standard: inadequate drug treatment defined as follows: one or more actions related to the drug treatment would have been medically justified and prioritised before the next regular visit but were not carried out, assessed in retrospect by two specialist physicians in consensus. Adequate drug treatment reflected the opposite, i.e. no additional action during the current visit related to the drug treatment would have been medically justified and prioritised before the next routine visit

The drug treatment of 48 (32%) patients was in consensus considered inadequate. For six (4%) of these patients, the assessment was related to the presence of at least one PIM listed in the studied indicator. The area under the ROC curve, for this indicator’s ability to classify the overall drug treatment as adequate or inadequate, was 0.56 (95% confidence interval (CI): 0.46–0.66) (Fig. 2, Table 4). With a 95% CIs passing 0.5, the indicator cannot discriminate between adequate and inadequate drug treatment.

**Table 4:** Number of PIMs according to the Swedish indicator ‘Drugs that should be avoided in older people unless specific reasons exist’^a^ and the indicator’s ability to classify drug treatment in older people as either adequate or inadequate^b^. The corresponding results using the number of drugs in the medication list (regular and as needed) as an indicator are presented for comparison

		‘Drugs that should be avoided in older people’^a^	Number of drugs (regular and as needed)
Median (range)		0 (0–2)	7 (0–20)
Area under ROC curve (95% CI)^c^		0.56 (0.46 to 0.66)	0.68 (0.59 to 0.77)
Optimal cut-off point^d^		≥ 1	≥ 6
Diagnostic measure at the optimal cut-off point	Sensitivity	0.27	0.79
	Specificity	0.84	0.54
	PPV	0.45	0.45
	NPV	0.71	0.85

*CI* confidence interval, *NPV* negative predictive value, *PIM* potentially inappropriate medication, *PPV* positive predictive value, *ROC* receiver operating characteristic

^a^Including long-acting benzodiazepines, drugs with anticholinergic action, tramadol, propiomazine, codeine, and glibenclamide

^b^Reference standard: adequate drug treatment was considered if no additional action during the current visit related to the treatment would have been medically justified and prioritised before the next routine visit, assessed in retrospect by two physicians in consensus. Inadequate drug treatment reflected the opposite, i.e. one or more actions related to the treatment would have been medically justified and prioritised but were not carried out

^c^The accuracy of the diagnostic tests was considered poor (0.60), fair (0.70), moderate (0.80), high (0.90), or near perfect (0.95) [20]

^d^Defined as the highest sum of sensitivity and specificity

## Discussion

This study shows that about one-fifth of primary care patients aged 75 years or older are treated with at least one PIM listed in the Swedish indicator ‘Drugs that should be avoided in older people’. The kappa values suggest strong agreement regarding the identification of patients with at least one PIM according to the studied indicator, but no agreement regarding physician assessments of their clinical relevance. In addition, the consensus assessments between the two physicians reveal that relatively few of these PIMs merit action from a medical perspective.

The area under the ROC curve shows that the studied indicator does not outperform chance in distinguishing between patients with adequate and inadequate drug treatment. An indicator based on the number of drugs in the medication list, on the other hand, performs better than chance. In this context, it must not be forgotten that the number of drugs reflects the burden of disease [22]. With greater complexity, the risk of overlooking something during a patient consultation is arguably higher. It may be worth noting that the optimal cut-off point in our study, to identify patients where action related to drug treatment could be medically justified and prioritised, is ≥ 6 drugs in the medication lists. Nonetheless, this threshold has low specificity.

The drugs included in the studied indicator have been determined by an expert panel to be generally unsuitable for older people [8]. However, like any other treatment, these medications require an individual assessment by the prescribing physician. Our results may thus illustrate the difference between what can be considered adequate/inadequate treatment at the population and individual levels, respectively; general treatment recommendations are based on an average individual, but no such individual exists in clinical practice. Still, the indicator may be a valuable tool to increase physicians’ attention to problematic medications and support them during the prescribing process. Indeed, the use of PIMs listed in the studied indicator has decreased in Sweden over time [14]. Nevertheless, our results suggest that there are still patients for whom these medications could be discontinued. Two drugs worth highlighting in this context are hydroxyzine for the treatment of anxiety and propiomazine for insomnia. In all cases, withdrawal of these drugs was considered medically justified, either immediately or in the long term.

In our study, 9% of the patients were prescribed drugs with anticholinergic action. The use of such drugs in those aged 75 years or older has increased over time [23]. Due to the side effects, however, such use may be particularly problematic. Indeed, the studied indicator, the Beers criteria [5], the EU(7)-PIM list [6], and the STOPP criteria [7] all include drugs with anticholinergic action (Table 5). Nevertheless, direct comparisons between criteria sets may be hampered by the fact that both drug-specific and diagnosis-specific criteria exist. Thus, knowledge of differences in criteria definitions is crucial when interpreting research results. Indeed, sets with drug-specific criteria may be suitable as screening tools and for prevalence studies, whereas complex diagnosis-specific criteria may be useful for prescribers in clinical practice and for educational purposes. Nevertheless, drug-specific criteria may have validity problems, supported by our finding that the studied indicator could not reflect the adequacy of drug treatment. Regarding complex diagnosis-specific criteria, on the other hand, both reliability and validity have been shown to be problematic as a substantial proportion have been reported not to be concordantly identified [24], and they also have limitations when it comes to their ability to reflect the adequacy of the drug treatment management [25].

**Table 5:** Drugs included in the Swedish indicator ‘Drugs that should be avoided in older people unless specific reasons exist’ and their presence in other internationally established PIM sets: the Beers criteria [5], the EU(7)-PIM list [6], and the STOPP criteria [7]

**Swedish indicator**	**Beers criteria**	**EU(7)-PIM list**	**STOPP criteria**
Long-acting benzodiazepines	Yes^a^	Yes	Yes^a,b^
Drugs with anticholinergic action	Yes	Yes	No^c^
Tramadol	Yes	Yes	No^d^
Propiomazine	No	Yes	No
Codeine	Yes^e^	Yes	No^e,d^
Glibenclamide	Yes	Yes	No^f^

*EU* European, *PIM* potentially inappropriate medication, *STOPP* Screening Tool of Older Persons’ Prescriptions

^a^Benzodiazepines in general, not restricted to long-acting benzodiazepines

^b^For ≥ 4 weeks and as part of diagnosis-specific criteria

^c^Included in diagnosis-specific criteria, e.g. drugs with potent anticholinergics/antimuscarinic effects in patients with delirium or dementia

^d^Included in diagnosis-specific criteria, e.g. long-term opioids for osteoarthritis

^e^Opioids as a drug class are included, not restricted to codeine

^f^Included in a diagnosis-specific criterion, i.e. sulphonylureas with a long duration of action (e.g. glibenclamide, chlorpropamide, and glimepiride) with type 2 diabetes mellitus

The complexity of medical assessments becomes evident with the level of inter-rater agreement between the physicians. In the original study, 167 (55%) out of 302 patients were assessed similarly as regards overall adequacy of drug treatment (kappa: 0.33) [16]. The primary care setting, where the physician considers the patient’s overall situation and where more than one drug and disease often have to be taken into account, could be a contributing factor. In the hospital setting, on the other hand, acute cases have to be managed; thus, more targeted medical priorities are likely to be made. Therefore, it may not be surprising that assessments of the appropriateness of drug treatment at hospital admission show high inter-rater agreement [26].

### Strengths and limitations

The main strength of this study is that it facilitates interpretation of results that are used in health care and research, both regarding the Swedish indicator per se and drug-specific PIMs in general. Indeed, although sometimes labelled as ‘inappropriate drugs’, they are in many cases not inappropriate for the specific patient when their individual circumstances are taken into account from a medical perspective. Furthermore, the results may illustrate the difference between adequately managed drug treatment according to general recommendations and what clinicians consider adequately managed treatment for a specific individual. However, the limited number of patients included in the study may be regarded as a limitation. Nevertheless, the consecutive inclusion of patients from two large primary care centres in both urban and rural settings [16, 17] may contribute to the generalisability of the results. Another limitation is that the data are from 2017; drug treatment changes over time, and three drugs within the current indicator have been withdrawn from the market in Sweden. Nonetheless, the entire criteria set of the Swedish indicator is still in use and has not been updated since 2017. Indeed, the studied indicator is incorporated in the electronic medical records of many primary health care centres and hospitals in Sweden, generating an alert during the prescribing process. Furthermore, almost all PIMs in the studied indicator are also present in the EU(7)-PIM list [6] and the Beers criteria [5]. A significant limitation is the absence of a reference standard to assess the quality of drug treatment. However, our reference standard relies on rigorous assessments of patients’ drug treatment and can be considered medically relevant, in particular as they were carried out first independently, preceded by the application of extensive explicit criteria sets, and then in consensus by two experienced physicians with relevant specialist expertise. Finally, it may be worth noting that the percentage of patients treated according to the studied indicator is higher in our study than in a national register study (19% versus 7%) [15]. Selection bias may contribute to this finding; our patients represented a consecutive sample attending a primary care centre. Furthermore, the medication lists in the current study were based on comprehensive information in the medical records, and drugs used as needed may not be captured when medication lists are estimated on filled prescriptions during a restricted time period [15, 27].

## Conclusion

This study shows that the Swedish indicator ‘Drugs that should be avoided in older people’ has strong reliability for the identification of PIMs in the medication list but does not validly reflect the adequacy of drug treatment. For most, but not all, patients in primary care, treatment with drugs listed as PIMs in the studied indicator may be medically justified and not prioritised to change during the medical visit.

### Supplementary Information

Below is the link to the electronic supplementary material.

Supplementary file1 (DOCX 22 KB)

## Data Availability

The database from this study is not publicly available owing to Swedish data protection laws. The data can be shared with authorised persons after they have applied and obtained approval from the Swedish Ethical Review Authority.

## Declarations

Some of the results presented in this article have previously been published in Swedish, in Läkartidningen 2023;120:23043 (PMID: 37964762).
