# Supplementary material for: Reliability and validity of the Swedish indicator ‘Drugs that should be avoided in older people’—an appraisal of a set of potentially inappropriate medications
Source: Eur J Clin Pharmacol. 2024 May 14;80(9):1285–93. doi: 10.1007/s00228-024-03700-x (PMC11303435; doi:10.1007/s00228-024-03700-x)
Supplement: Supplementary file 1 — Supplementary file1 (DOCX 22 KB) [file 228_2024_3700_MOESM1_ESM.docx]

**Supplementary material**

**To:** Parodi López N, Svensson SA, Lönnbro J, Hoffmann M, Wallerstedt SM. Reliability and validity of the Swedish indicator “Drugs that should be avoided in older people” ‒ an appraisal of a set of potentially inappropriate medications. Eur J Clin Pharmacol. 2024.

**Corresponding author:** Naldy Parodi López, Department of Clinical Pharmacology, Sahlgrenska University Hospital, Gothenburg, Sweden. E-mail: naldy.parodi.lopez@gu.se.

**Swedish set of indicators for quality of drug therapy in the elderly**

Summarised from specific indicators in:

National Board of Health and Welfare. Indicators for appropriate pharmacotherapy in older people [Indikatorer för god läkemedelsterapi hos äldre]. 2017-6-7.

1. Drug-specific indicators
   1. **Drugs that should be avoided unless specific reasons exist**

**Long-acting benzodiazepines**

**Drugs with anticholinergic effects**

**Tramadol**

**Propiomazine**

**Codeine**

**Glibenclamide**

- 1. Drugs for which a correct or current indication is often lacking or unclear

Cyclooxygenase (COX) inhibitors

Paracetamol

Opioids

Antipsychotic drugs

Proton pump inhibitors (PPI)

Digoxin

Loop diuretics

Selective serotonin reuptake inhibitors (SSRI) and other antidepressants

Low-dose acetylsalicylic acid (ASA)

Statins

Hypnotics

Drugs for urinary frequency and incontinence

Allopurinol

- 1. Inappropriate regimen

Hypnotic, regular use >1 month without review

COX inhibitor, regular use >2 weeks without review

Systemic steroid, regular use >1 year without review

Antiepileptic >1 year without review

Antipsychotic >3 months without review

PPI >2 months without review

Drug for urinary frequency and incontinence >2 months without review

Benzodiazepine, regular use >1 month without review

- 1. Inappropriate dosing

Low-dose ASA >75 mg

Haloperidol >2 mg/day

Risperidone >1.5 mg/day

Oxazepam >30 mg/day

Zopiclone >7.5 mg/day

Citalopram >20 mg/day

Escitalopram >10 mg/day

- 1. Polypharmacy

≥2 drugs from the same ATC group or with the same mechanism of action

≥3 psychotropic drugs

- 1. Interaction alerts where the recommendation is to avoid the drug combination

Category D according to the Swedish national interaction database (*Janusmed*)

- 1. Drugs and renal function

Estimated GFR calculated at least annually for drugs where dose adjustments need to be considered according to renal function (rational use)

- 1. Drugs and specific symptoms

Review of drugs with orthostatic properties in case of symptomatic orthostatic hypotension (rational use)

Review of drugs with fall-risk increasing properties in case of increased risk of falls (rational use)

Review of drugs affecting cognition in case of cognitive deterioration (rational use)

- 1. Psychotropic drugs

Oxazepam (rational use)

Zopiclone (rational use)

1. **Diagnosis-specific indicators**
   1. Hypertension
      1. Rational use

Angiotensin-converting enzyme (ACE) inhibitor or angiotensin II receptor blocker (ARB) and/or dihydropyridine calcium channel blocker and/or thiazide diuretic

ACE inhibitor or ARB in concurrent diabetes and renal impairment

- - 1. Potentially inappropriate use

Combination of beta blocker and verapamil or diltiazem, without atrial fibrillation Verapamil or diltiazem in concurrent systolic heart failure

- 1. Chronic ischemic heart disease
     1. Rational use

Low-dose ASA or clopidogrel or oral anticoagulant

Beta blocker

Statin

- - 1. Potentially inappropriate use

ASA >75 mg/day

Diltiazem or verapamil in concurrent systolic heart failure

Diltiazem or verapamil combined with beta blocker without atrial fibrillation

- 1. Congestive heart failure
     1. Rational use

Diagnosis confirmed with echocardiography

ACE inhibitor or ARB in concurrent systolic heart failure

ACE inhibitor/ARB and beta blocker in concurrent systolic heart failure

Add-on aldosterone antagonist (spironolactone or eplerenone) if reduced ejection fraction (≤35%) and congestive heart failure symptoms (NYHA II-IV) despite full baseline therapy

- - 1. Potentially inappropriate use

Digoxin without atrial fibrillation

Potassium if treated with potassium-sparing drugs

COX inhibitors

Verapamil or diltiazem

Disopyramide, propafenone, flecainide, dronedarone or sotalol

- 1. Chronic obstructive pulmonary disease
     1. Potentially inappropriate use

Beta-2 agonist, inhaled anticholinergic or inhaled steroid without demonstrated beneficial effect

Oral beta-2 agonist

Regular treatment with short-acting beta-2 agonist inhaler

Long-term therapy with oral steroid

Acetylcysteine

Non-selective beta blocker

≥1 inhaler without review of inhalation technique and inhalation capacity

- 1. Diabetes mellitus type 2
     1. Potentially inappropriate use

Metformin if eGFR <30 ml/min

Glibenclamide

Sulfonylureas or repaglinide if eGFR <30 ml/min

Pioglitazone, acarbose, glucagon-like peptide-1 (GLP-1) agonist, or sodium-glucose transport protein 2 (SGLT-2) inhibitor

- 1. Gastroesophageal reflux disease and ulcer-rational use
     1. Rational use

Eradication therapy in confirmed H. Pylori

- - 1. Potentially inappropriate use

COX inhibitor or ASA without prophylaxis against ulcer in those with a past history of ulcer

Steroid for systemic use combined with COX inhibitor or ASA without prophylaxis against ulcer in those with a past history of ulcer

Omeprazole/esomeprazole in combination with citalopram/escitalopram

Long-term PPI without review

- 1. Urinary tract infection
     1. Rational use

Nitrofurantoin or pivmecillinam in acute cystitis

- - 1. Potentially inappropriate use

Nitrofurantoin if eGFR <40 ml/min

Quinolone in urinary tract infection without fever

- 1. Pain
     1. Rational use

Tricyclic antidepressant or gabapentin in neuropathic pain

- - 1. Potentially inappropriate use

COX inhibitor as first line therapy

Opioid as first line therapy

Regular use of a COX inhibitor

High dose of a COX inhibitor

Tramadol

Codeine

- 1. Dementia
     1. Rational use

Acetylcholinesterase inhibitor or memantine

- - 1. Potentially inappropriate use

Acetylcholinesterase inhibitor or memantine without a confirmed diagnosis

Acetylcholinesterase inhibitor or memantine in frontotemporal or vascular dementia

Acetylcholinesterase inhibitor or memantine despite lack of confirmed beneficial effect

Acetylcholinesterase inhibitor combined with other drug with anticholinergic effects

Drugs with anticholinergics effects

Sedation with anxiolytic or sedative drugs

Antipsychotic drug for other indications than severe psychotic symptoms or severe aggressive behaviour

- 1. Depression

2.10.1. Rational use

Effect of antidepressant reviewed within 3 months after initiation

2.10.2. Potentially inappropriate use

Antidepressant without confirmed depression diagnosis

Citalopram >20 mg/day

Escitalopram >10 mg/day

- 1. Insomnia

2.11.1. Rational use

Zopiclone

2.11.2. Potentially inappropriate use

Long-acting benzodiazepine

Propiomazine

Hydroxyzine, alimemazine or prometazine

- 1. Atrial fibrillation/stroke prophylaxis

2.12.1. Rational use

Anticoagulant

2.12.3. Potentially inappropriate use

ASA or other platelet inhibitor

- 1. Osteoporosis

2.13.1. Rational use

Antiresorptive drugs within 12 months after first fragility fracture

2.13.2. Potentially inappropriate use

Calcium and vitamin D in monotherapy 6-12 months after first fragility fracture
